# Legacy of Pre-Disturbance Spatial Pattern Determines Early Structural Diversity following Severe Disturbance in Montane Spruce Forests

**DOI:** 10.1371/journal.pone.0139214

**Published:** 2015-09-30

**Authors:** Radek Bače, Miroslav Svoboda, Pavel Janda, Robert C. Morrissey, Jan Wild, Jennifer L. Clear, Vojtěch Čada, Daniel C. Donato

**Affiliations:** 1 Department of Forest Ecology, Faculty of Forestry and Wood science, Czech University of Life Sciences, Prague, Czech Republic; 2 Institute of Botany, The Czech Academy of Sciences, Průhonice, Czech Republic; 3 Department of Applied Geoinformatics and Spatial Planning, Faculty of Environmental Sciences, Czech University of Life Sciences, Prague, Czech Republic; 4 Washington State Department of Natural Resources, Olympia, Washington, United States of America; Lakehead University, CANADA

## Abstract

**Background:**

Severe canopy-removing disturbances are native to many temperate forests and radically alter stand structure, but biotic legacies (surviving elements or patterns) can lend continuity to ecosystem function after such events. Poorly understood is the degree to which the structural complexity of an old-growth forest carries over to the next stand. We asked how pre-disturbance spatial pattern acts as a legacy to influence post-disturbance stand structure, and how this legacy influences the structural diversity within the early-seral stand.

**Methods:**

Two stem-mapped one-hectare forest plots in the Czech Republic experienced a severe bark beetle outbreak, thus providing before-and-after data on spatial patterns in live and dead trees, crown projections, down logs, and herb cover.

**Results:**

Post-disturbance stands were dominated by an advanced regeneration layer present before the disturbance. Both major species, Norway spruce (*Picea abies*) and rowan (*Sorbus aucuparia*), were strongly self-aggregated and also clustered to former canopy trees, pre-disturbance snags, stumps and logs, suggesting positive overstory to understory neighbourhood effects. Thus, although the disturbance dramatically reduced the stand’s height profile with ~100% mortality of the canopy layer, the spatial structure of post-disturbance stands still closely reflected the pre-disturbance structure. The former upper tree layer influenced advanced regeneration through microsite and light limitation. Under formerly dense canopies, regeneration density was high but relatively homogeneous in height; while in former small gaps with greater herb cover, regeneration density was lower but with greater heterogeneity in heights.

**Conclusion:**

These findings suggest that pre-disturbance spatial patterns of forests can persist through severe canopy-removing disturbance, and determine the spatial structure of the succeeding stand. Such patterns constitute a subtle but key legacy effect, promoting structural complexity in early-seral forests as well as variable successional pathways and rates. This influence suggests a continuity in spatial ecosystem structure that may well persist through multiple forest generations.

## Introduction

Large, severe disturbances are native to many temperate forests, but are increasing in frequency and extent in many forest landscapes, a trend predicted to continue with future climate change [1–3]. These events profoundly influence ecosystem structure and function, generating wide variability in ecological responses relative to smaller, less severe disturbances [4] and, thus, greater uncertainty for stand development and management [5]. An emerging theme in forest ecology is understanding the role of spatial heterogeneity relative to disturbances, biological legacies, and other factors influencing stand development and biodiversity (e.g. [6–8]).

A key factor influencing the stability of ecosystem pattern through disturbance is the presence of biological legacies, defined as biologically-derived elements of the pre-disturbance ecosystem that carry over into the next stand [9]. The most commonly studied examples include standing and down dead trees, pits and mounds from uprooted trees, soil organic matter, sexually mature live trees, understory seedling and sapling banks (advance regeneration), and vegetatively reproducing parts and seed banks [9]. The role of advance regeneration in driving succession following canopy-removing disturbances (via abundance and composition of the surviving cohort) has been well established across several forest types [10–12]. Rarely studied, however, is how pre-disturbance forest spatial patterns, including advance regeneration and the former overstory, influence the structure of post-disturbance stands. Although disturbances may be severe enough to erase pre-disturbance structural patterns [5,13], recent studies show persistent horizontal patterns after disturbances [6,8], and that such fidelity of pattern can persist for more than two centuries [14–15]. However, the drivers underlying this continuity of pattern and the consequences for early-seral stand structure and development are poorly understood.

The legacy influence of advance regeneration, and the factors that structure its spatial pattern, are especially important after disturbances that cause near complete overstory removal. For example, Norway spruce (*Picea abies* (L.) Karst.) montane forests of Central Europe, where wind storms and bark beetle outbreaks have recently caused total overstory dieback across thousands of hectares [16–19], regenerate primarily from an understory seedling and sapling bank, and post-disturbance seedling germination is a relatively minor component of regeneration [20–21]. Patterns of advance regeneration in mature stands are influenced by overstory distribution through seed distribution and light availability for understory trees [22], and ground-layer vegetation which can act as a strong filter on succession of advance regeneration both before and after disturbance [23]. Deadwood such as snags, stumps, down logs, and their immediate vicinity, are also important to advance regeneration and survival by providing favourable germination sites and microenvironments for Norway spruce [24]. Thus, pre-disturbance interactions between mature overstories, microsite availability, and understory distribution and growth may effectively determine the stand structure following severe canopy-removing disturbance.

Canopy-opening disturbances positively influence the diversity of organisms that peak in newly-opened stands (e.g. fresh deadwood specialists) as well as those associated with more developed early-seral vegetation for subsequent decades [25–29]. However, open habitats (e.g. sun-exposed large deadwood) are expected to diminish after tree canopy closure; thus, variable post-disturbance pathways that include protracted early-seral stages and promote the survival of early-seral dependent species can maintain greater biodiversity.

Identifying the influence of pre-disturbance spatial pattern on post-disturbance stand structure has been limited by a lack of spatially explicit data monitored through a disturbance event. Most studies have used only post-disturbance data to reconstruct some components of the prior stand [8,11,30]. Monitoring spatial pattern through time, even in a limited number of plots, can yield a unique data set that gives more accurate insight into stand dynamics [31]. After two large research plots in a montane spruce forest with mapped stand structure data were impacted by a major (100%) canopy-removing windstorm and bark beetle outbreak, we re-measured and mapped the plots. We used these spatially explicit data to evaluate how the structural pattern of regeneration relates to pre-existing microsites, overstory pattern, and understory vegetation, factors known to influence stand development pathways. Tree species composition, spatial distribution, height, and height heterogeneity were compared before and after disturbance with the following objectives: (1) to determine how pre-disturbance spatial pattern acts as a legacy to influence post-disturbance spatial structure and; (2) to evaluate how the fidelity of horizontal pattern influences the structural diversity within the early-seral stand.

## Methods

### Study site

This study was conducted in a Norway spruce forest situated in the Bohemian Forest (Šumava in Czech; 48°47′N, 13°49′E) located in the southwest of the Czech Republic. The Administration of the National Park and Protective Landscape Area of Šumava granted the permission for data collection. The region was established as a nature reserve in 1933 before becoming part of the Šumava National Park in 1991. Study plots are located between 1220–1270 m a.s.l., with a north aspect and gentle slope (≤8°). Total annual precipitation is ca. 1400 mm and mean annual temperature is approximately 4°C [32]. Snow cover usually persists from November to early May and the maximum snow depth is about 2 m. The bedrock is coarse-grained granite. Plant communities are classified as *Athyrio alpestris-Piceetum* [33] with abundant Alpine-lady fern (*Athyrium distentifolium* Tausch ex Opiz) undergrowth.

Present day forest composition and structure is the legacy of the historical disturbance regime, characterized by infrequent large-scale, moderate-severity (non-stand-replacing) disturbances combined with frequent low-severity events during the last 300 years [34–35]. Before the most recent severe disturbance, the forest was dominated by spruce trees with an unbalanced bimodal height distribution [36]. The age distribution of trees was also bimodal; with numerous trees older than 250 years, almost no recruitment during the first half of the 20th century, then a more recently established advance regeneration layer [35]. Overstory canopy cover was <30% on both study plots and regeneration was dominated by spruce and rowan (*Sorbus aucuparia* L.). Sapling (50–200 cm tall) density was 1095 and 253 individuals per hectare for spruce and rowan, respectively. Spruce seedling (<50 cm tall) density was 4 400 individuals per hectare [36]. A bark beetle (*Ips typographus* L.) outbreak between 1996 and 1999 resulted in a mosaic of dead tree patches, each up to one hectare. This was followed in 2007 by a winter storm (‘Kyrill’) that caused severe and widespread uprooting of mature spruce trees. A subsequent bark beetle outbreak resulted in mortality of all canopy spruce trees in 2008.

### Data collection

In 2005, two permanent sampling plots (100×100 m) were established in the study area. Plots were selected to minimize the inclusion of trees recently killed by the earlier bark beetle disturbance (between 1996 and 1999) and to avoid the occurrence of specific site conditions (e.g. stream corridors, rock outcrops, or evidence of logging). Thus, sample plots were initially in relatively undisturbed, unmanaged old growth forest.

Electronic and laser measuring devices (Field-Map^®^, Monitoring and Mapping Solutions, Ltd.; www.fieldmap.cz) were used to map and field-tag all live individuals of spruce >50 cm height and of rowan >30 cm height, all standing deadwood with diameter at ground level >10 cm, and down logs with large end diameter >10 cm and >2 m length. Species and height were recorded for each tree. Down logs were classified into 4 categories according to side vegetation cover (the vegetation growing along a log and/or rising over the log): (1) up to 5% of the log covered; (2) 6 to 25% covered; (3) 26 to 50% covered and (4) >50% covered. The crown projection of trees taller than 4 m was recorded by a minimum of five radii extending to the edge of the crown. Spruce individuals below 50 cm (seedlings) were sampled in 25 5×5 m subplots located at every other intersection and end of a 10 m grid overlain on the plot. Seedlings were measured and classified into one of five 10 cm height classes. The coverage of all vascular plants was estimated (to the nearest 5%) in each 10×10 m grid cell and within each 5×5m subplot.

Following the complete overstory mortality generated by the windstorm (only ~10% of canopy trees were uprooted) and ensuing beetle outbreak (~90% canopy trees were killed), a post-disturbance inventory of the stem-mapped plots was conducted in 2009. All individuals were identified and recorded as; survived, damaged (breakage, bending, drying, uprooting) or dead. The reason for tree mortality was subsequently identified as: bark beetle attack; wind uprooting; falling caused by mechanical forcing (by another mature tree, snag or live uprooted tree); uprooting from deadwood microsite; competition; ungulate damage; and other (e.g. snow). On plot 2, all newly recruited (post-disturbance) saplings above 50 cm (spruce) and 30 cm (rowan) were also spatially mapped.

### Data analysis

#### Pre-disturbance

For the purpose of analysis, saplings were defined as individuals ≤2 m tall, and canopy trees were those >25 m in height. To identify differences in spatial pattern of small versus large sapling regeneration, saplings were divided into two groups of approximately equal abundance using a boundary value of 90 cm in height. Individuals between 2 and 25 m tall were not included in the analysis regarding influence of canopy trees on regeneration. All analyses were conducted using R statistical software (v.2.15.2; R Development Core Team 2009). Tree spatial patterns were analysed using pair correlation function (*pcf*), estimated by smoothing with Epanechnikov kernel and Ripley’s isotropic edge correction implemented in spatstat R-package [37–38]. Univariate pcf were compared to envelopes to assess spatial relations within the advance regeneration cohort. Envelopes were constructed by removing the four highest and four lowest values of 199 random simulations of a complete spatial randomness (CSR) null model. The relations among different tree groups (i.e. among saplings of different species, saplings and canopy trees, snags and stumps) were assessed using a bivariate form of *pcf* [37–38]. To test the independence of two spatial patterns we constructed null model by random shifting of all position of one group, while keeping a fixed position of the second group [39–40]. The envelopes were than constructed similarly as by univariate *pcf*.

To examine the relationship between sapling density and potential overstory and understory competition, as indicated by canopy cover and Alpine-lady fern cover, respectively, the *rhohat* procedure [41] was implemented using spatstat R-package [38]. Sapling density was plotted as a function of canopy cover and Alpine-lady fern cover in each 10×10 m grid cell. Canopy cover within the grid cells was defined as 100% minus the area without crown projection. The cover of Alpine-lady fern was used for analysis because it is the most abundant vascular plant in this locality and reacts positively to an increase in light availability [42]. The *rhohat* procedure was also used to evaluate the dependency of spruce sapling establishment on down logs, with distance from the nearest long axis of a down log used as a covariate. Spruce sapling establishment associated with individual logs was then compared according to the amount of side vegetation cover.

Finally, we assessed how the occurrence of regeneration of different height classes and the heterogeneity versus homogeneity of sapling heights varied by patch type within the stands (high or low canopy cover; high or low Alpine-lady fern cover) using a principal component analysis (PCA). The PCA were based on a subplot-by-height class matrix, with the regeneration densities occupying matrix cells. Spruce seedling and sapling densities were log-transformed and scaled in analyses of both seedling (5×5 m) subplots and sapling (10×10 m) subplots. To describe the main gradients, canopy and Alpine-lady fern cover variables (with r^2^>0.1 and p<0.001) were passively projected into ordination space using the *envfit* function with 999 permutations [43]. This method produces vectors that represent the most rapidly changing direction for a given variable and have lengths proportional to the strength of the correlation between variables and the ordination. To display the position of regeneration characteristics in ordination space, the arrows representing regeneration density (trees ha^-1^), height homogeneity (kurtosis of height distribution) and height heterogeneity (interquartile range of height distribution) were also overlain on the ordination space. All independent variables are listed in S1 Table.

#### Post-disturbance

To determine whether disturbance-generated changes occur in a spatially correlated way over pre-disturbance univariate patterns of trees, bivariate *pcf* between killed and newly recruited individuals above 50 cm and survived individuals were used. To test the spatial independence of survived and newly recruited spruce we constructed a null model by random shifting of all position of survived trees, while keeping a fixed position of new recruits [39–40]. The spatial independence of demographic events like mortality and recruitment within a fixed pattern approach were tested using random labelling of either killed and survived or survived and newly recruited individuals for each simulation [39–40]. The envelopes for all *pcf* tests were constructed by removing the four highest and four lowest values of 199 simulations [38]. To evaluate changes in spruce-free patch size (gaps in the pattern), the ‘empty space distance F-function’ with Kaplan-Meier edge correction was used in the spatstat package [38]. The empty space distance F is the distance from an arbitrary fixed location to the nearest point of the pattern.

To test the difference in increase between spruce and rowan saplings, Pearson's Chi-squared test with Yates' continuity correction was used. To compare the height increment among spruce and rowan saplings, an ANCOVA and associated F-test was used with 2005 height measurements as a covariate and species as an explanatory variable.

## Results

### Pre-disturbance

#### Spatial pattern of advance regeneration

**Fig 1:**
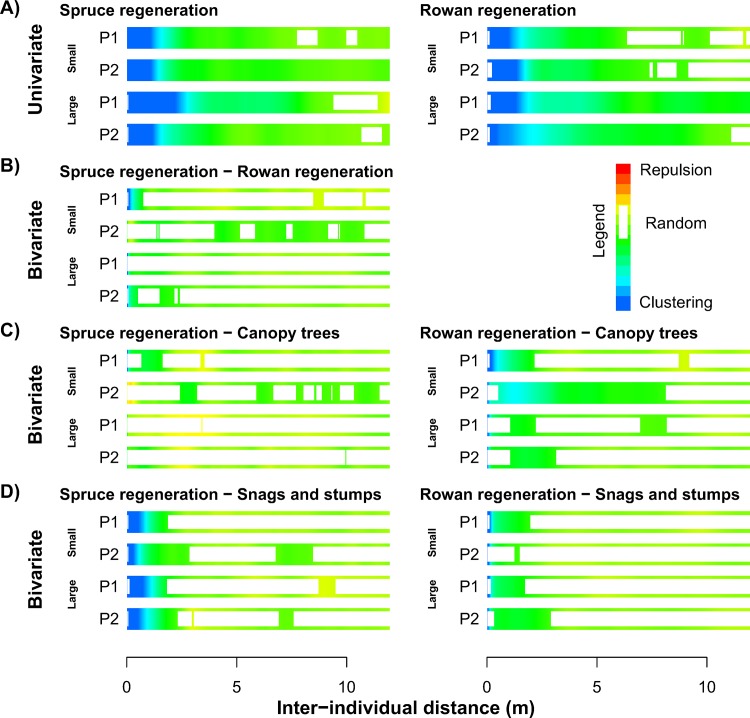
Spatial patterns of regeneration. A) Spatial patterns of saplings (small: 50–90 cm, large: 90–200 cm); B) spatial relationship between spruce and rowan saplings; C) spatial relationship between saplings and canopy living trees and D) spatial relationship between saplings and snags or stumps evaluated with univariate and bivariate pair correlation function. Both strongly self-aggregated spruce and rowan saplings are clustered to canopy (>25 m) trees, snag and stumps.

When individually analysed (within species), both spruce and rowan regeneration were spatially aggregated; strongly at distances of up to 2–3 m and slightly to a distance of 8–10 m (Fig 1A). The bivariate spatial pattern of spruce and rowan regeneration indicates weak aggregation or randomness at most scales and this pattern is variable between plots (Fig 1B). Rowan regeneration exhibited clustering with canopy spruce trees at distances between 1–3 m, particularly for smaller saplings (Fig 1C). Spruce regeneration is more spatially independent of canopy trees, especially for tall saplings (Fig 1C). Both rowan and especially spruce regeneration were clustered with pre-disturbance snags and stumps at distances up to approximately 2 m (Fig 1D).

### Structure of advance regeneration in relation to canopy and understory cover

**Fig 2:**
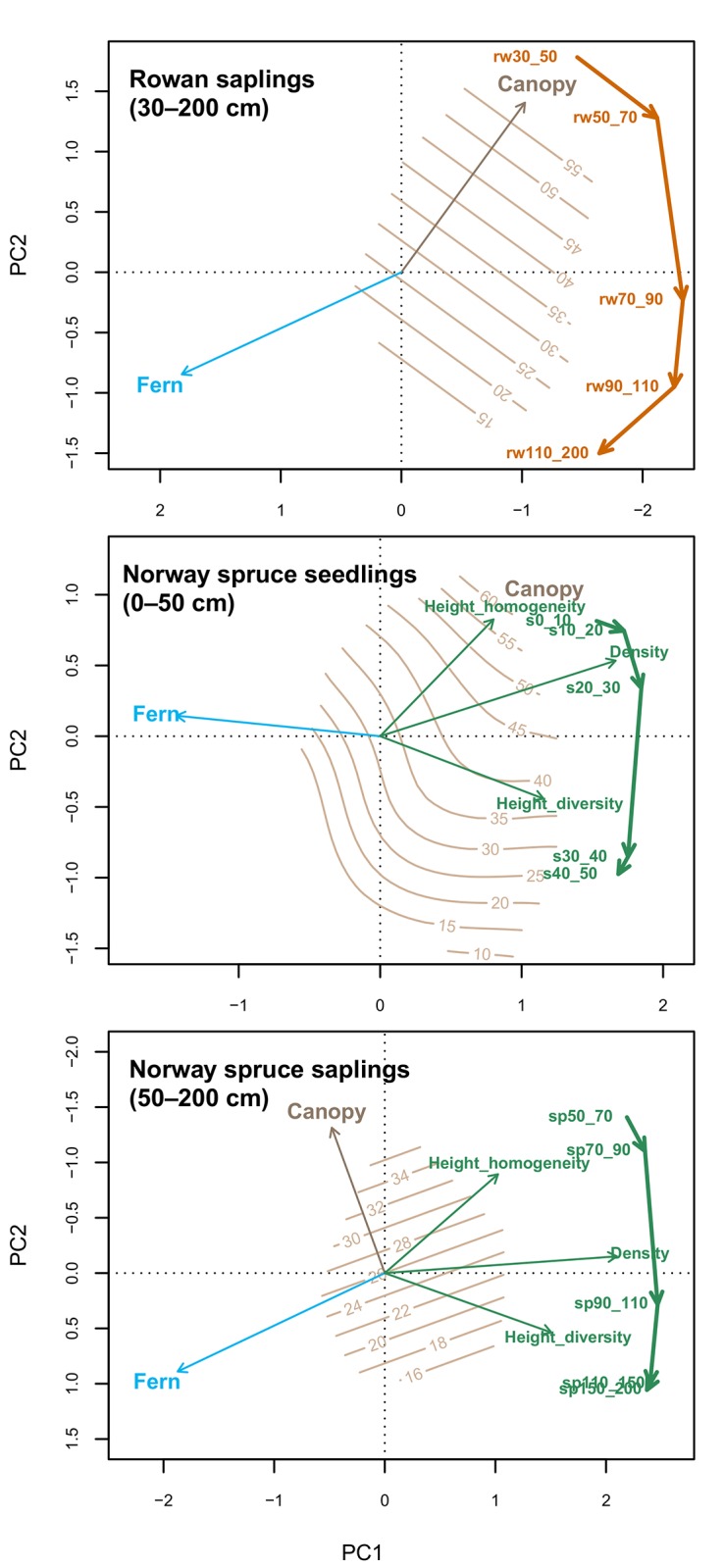
Ordination biplots of rowan sapling, spruce seedling, and spruce sapling densities in different height classes. Regeneration densities are represented by text (indicating height in cm) and arrows show the direction from smaller to larger height classes of saplings. The passively projected environmental variables (Alpine-lady fern and canopy cover) are represented by blue and brown arrow. The passively projected spruce regeneration density, height homogeneity and height heterogeneity are overlain. Brown isolines show the gradient in the canopy cover.

The density of both spruce and rowan advance regeneration exhibited a unimodal ‘hump’ distribution with respect to both overstory canopy cover and Alpine-lady fern cover (S1 Fig). Reduced sapling density is associated with highly closed tree canopies, and also under very open canopies, where Alpine-lady fern was abundant. PCA also indicates that advance regeneration of spruce and rowan was strongly spatially limited by patches with dense herbaceous cover formed by Alpine-lady fern (Fig 2). Height class trajectories and canopy cover exhibited a negative relationship for spruce and rowan regeneration (tall saplings are associated with more open conditions). Spruce saplings exhibited higher density values near logs with lower levels of competition, particularly at distances <2 m (S2 Fig). However, spruce sapling density was low near logs with high vegetation cover, and they were often limited to microsites directly on the deadwood (0 m).

### Post disturbance

#### Species composition and height structure

**Fig 3:**
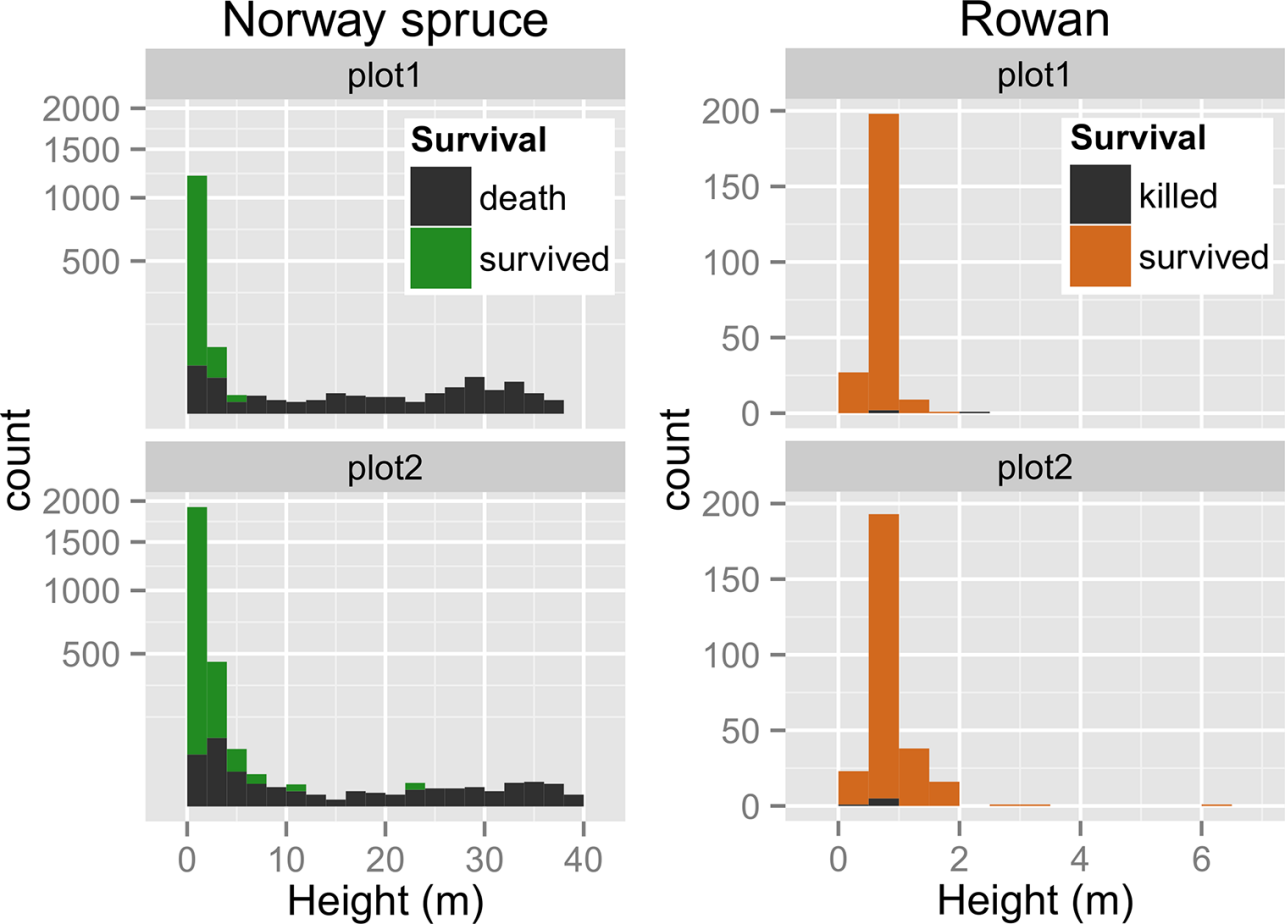
Histograms of individual tree heights categorized by species and plot before disturbance (2005). Surviving and dead individuals after disturbance are marked in colour. The y-axes for spruce histogram were square root-transformed.

After the 2007 windstorm, virtually all canopy trees were killed; some by the initial windstorm but the majority by subsequent bark beetle outbreak. The height threshold for survival of spruce trees through the disturbance was approximately 2–4 m (Fig 3). The disturbance effect on existing regeneration was relatively low (S2 Table). The only notable disturbance-related injury was damage and death of spruce saplings by another large live tree falling during the windstorm, whether due to the whole trunk or just branch fall. Rowan remained mostly unaffected; only 5 individuals died, and these were a result of live tree fall (S2 Table). The number of newly recruited spruce saplings, above 50 cm, was significantly higher than the number of newly recruited rowan saplings (Plot 2: *χ* ^*2*^ = 8.38, *p* = 0.004), at 39% (535 new individuals) and at 26% (70 new individuals), respectively. Rowan post-disturbance height increment (average [± standard deviation]: 43.0[±44.5] cm) was significantly higher than spruce (12.7[±39.6] cm).

#### Spatial pattern

**Fig 4:**
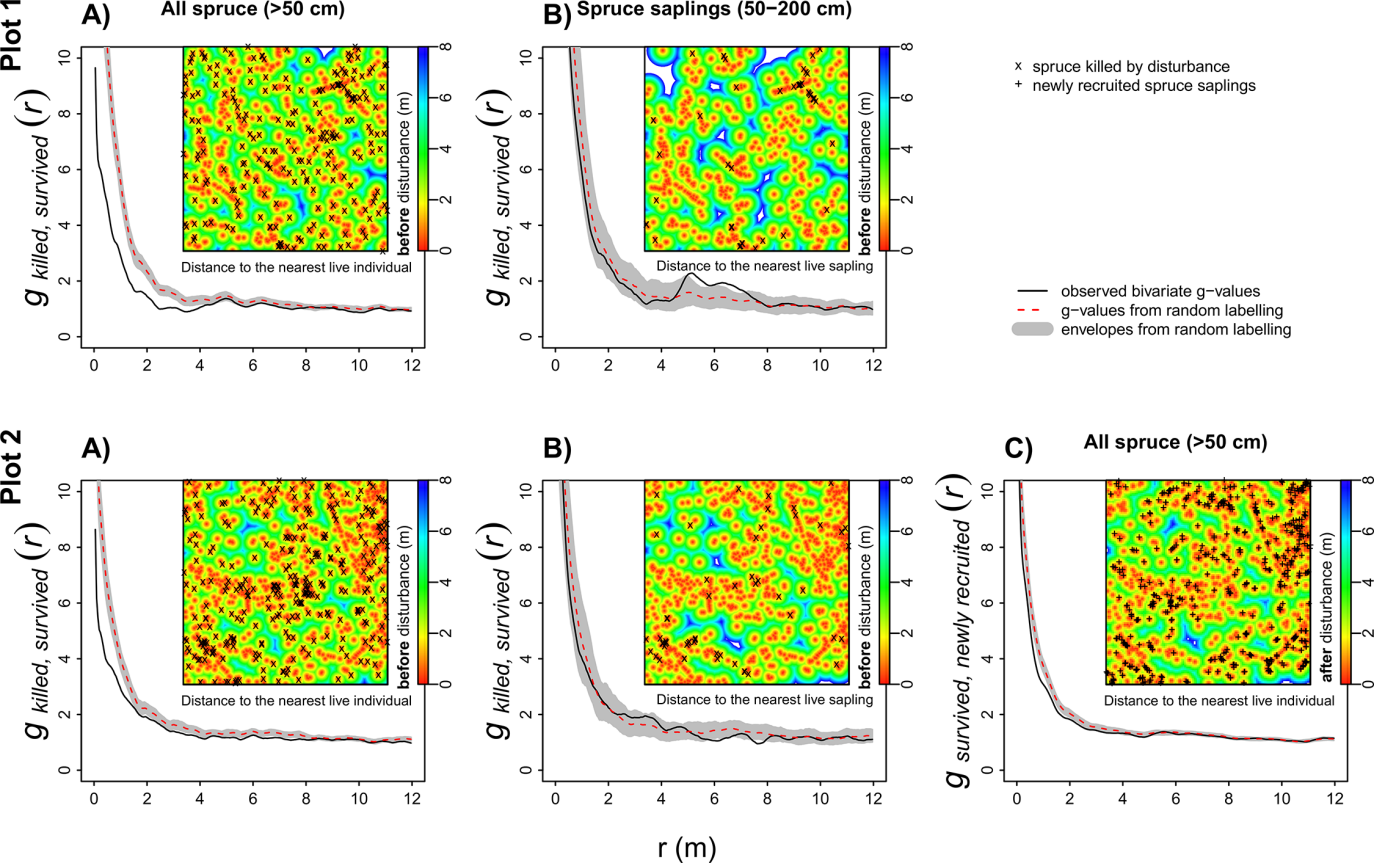
Mortality and recruitment spatial pattern. Bivariate spatial pattern between disturbance-killed and survived individuals of; (A) all pre-disturbance recruited spruce, (B) pre-disturbance recruited spruce saplings and (C) the bivariate pattern between all survived and newly recruited spruce above 50 cm. Positions of spruces killed by disturbance (A, B) or newly recruited spruces (C) are marked in each subplots. Spruce-free patches (gaps) before disturbance (A, B) and after disturbance (C) are stressed by colour spectrum that represents the distance to the nearest live individual in meters.

**Fig 5:**
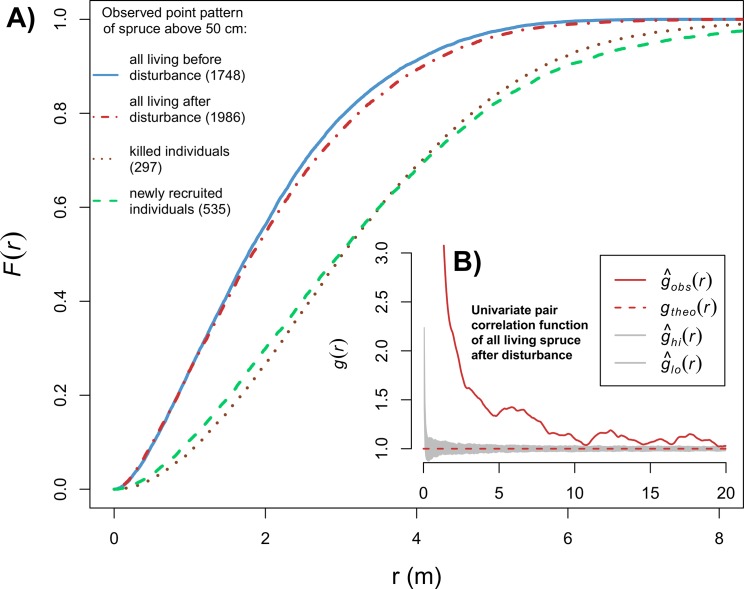
Distribution of empty space function (*F(r)*) from spruce point pattern. Point pattern is formed by living before and living after disturbance, killed by disturbance and newly recruited spruces above 50 cm. The numbers of individuals are specified in brackets. The evaluation of post-disturbance living spruce spatial pattern is shown in subplot using univariate pair correlation function and CSR null model.

The disturbance-related mortality among all spruce individuals above 50 cm (including canopy trees) was not random within fixed pre-disturbance pattern. The bivariate *pcf* suggested partial segregation of killed individuals from survived individuals at finer scales (up ~8 m) (Fig 4A). But disturbance-related mortality among saplings within fixed pre-disturbance spatial pattern did not significantly differ from the random mortality null model (Fig 4B). This indicates that canopy trees, which have the highest mortality rate (Fig 3), were less aggregated to saplings than saplings were among themselves. New spruce trees were found to establish close to survived individuals, more than predicted by the null model based on random shifting of new recruits (S3 Fig). However, the process that distributed the label (survivor or new recruit) within the fixed pattern was not random. Slight negative departures (to a distance of 3 m) from the random labelling null model show slight segregation between survivors and new recruits (Fig 4C). Consequently, the post-disturbance spruce-free patches were in the same locations (compare gaps on subplots Fig 4, Plot 2) and were slightly larger than spruce free-patches formed before disturbance, despite the fact that the number of newly recruited spruce above 50 cm was greater than the number of killed individuals (Fig 5A). The post-disturbance living spruce spatial pattern remains strongly aggregated (Fig 5B).

## Discussion

Detailed analyses of horizontal and vertical forest structure pre- and post-disturbance allowed us to reveal some of the drivers of regeneration pattern and revealed the potential precursors of early-seral structural diversity. The spatial pattern of regeneration before disturbance was driven mainly by canopy closure, the density of herb layer vegetation, and spatial aggregation among various tree life stages. After the disturbance, all canopy trees were killed, but the disturbance had little impact on advance regeneration; variability of regeneration density and height was similar pre- and post-disturbance. Overall horizontal post-disturbance spatial pattern was maintained primarily by the strong spatial aggregation among surviving spruce and rowan regeneration, killed canopy spruce trees, and pre-existing snags and stumps. The locations of low-density and high-density tree patches remained largely the same through the disturbance.

### Pre-disturbance spatial pattern influences post-disturbance stand horizontal and vertical structure

The spatial pattern of living trees after severe bark beetle disturbance was almost exclusively influenced by the pre-disturbance state. Pre-disturbance advance regeneration was resistant to disturbance and had high survival, and newly recruited individuals established proximal to existing individuals. Although the disturbance dramatically reduced the height structure through total mortality of the canopy layer, post-disturbance vertical structure still partly reflected the pre-disturbance structure. The memory of vertically structured stands is incorporated implicitly in to the localized severity of a disturbance of otherwise similar intensity; i.e. larger trees are more susceptible to disturbance [44–45]. Forests with more developed vertical structure retain the pattern of the previous state to a greater degree, because the smaller trees survive the disturbance; homogeneous stand structures typical of managed forests have lower vertical diversity, thus they tend to be less resistant and resilient [46]. Vertical heterogeneity was further enhanced by the diversity of height growth responses of surviving regeneration (coefficient of variation = 3.1) and the persistence of spruce-free patches, which are large enough to allow the further establishment of younger individuals in the future and further diversify the age and height structures. Suzuki et al. [6] attributed the observed long-term structural heterogeneity in part to the variability of individual tree growth associated with different patterns of regeneration survival and distribution. We know of no other studies that have examined the pre- and post-disturbance spatial patterning of regeneration, however, several studies have examined post-disturbance tree distribution (e.g. [8,47–49]).

The spatial aggregation among surviving spruce and rowan regeneration, killed canopy spruce trees, and pre-existing snags and stumps (Fig 1) confirm the existence of a strong positive overstory to understory neighbourhood effect [8,50]. The positive neighbourhood effect of overstory on spruce recovery is mediated by; (1) direct regeneration of spruce on deadwood or in vicinity of stems [8,24] and (2) reducing competition with understory plants due to unfavourable light conditions under the canopy [42]. Suitable microsites for young seedling establishment of both species occurred in patches with higher levels of canopy cover [42,51–52]; in the presence of dense undergrowth vegetation, even the most suitable microsites (deadwood) are only sparsely populated by spruce saplings (S2 Fig).

The relationship between regeneration density and undergrowth plant and canopy cover suggests a disturbance-activated positive neighbourhood effect [50]. Although Norway spruce evolved various adaptations for an ‘advance regeneration system’ including adventitious root formation [53], the period of understory persistence is limited and probably does not exceed 50 years [24,54]. Spruce regeneration density tended to be low in areas of moderate canopy cover (20–55%). For seedlings, high competition pressure within the herb layer makes establishment and survival difficult over long time periods [23,42,55]. Those that do survive to sapling size have increasing light requirements with increasing size [22]. Without canopy disturbance, saplings at this stage are increasingly subject to overwintering injuries [56], herbivore pressure [57], and allelopathy and resource competition by herb layer [24,58]; thus, areas of moderate canopy cover may have low or no spruce regeneration. A disturbance-activated positive neighbourhood effect is evident with the release of residual seedlings, which improves height growth through increased light availability.

### Severe bark beetle disturbance preserved forest structural complexity and composition

The severe bark beetle disturbance reduced vertical structure within these stands; the wind and beetle disturbance strongly selected against overstory spruce, but the legacy of advance regeneration also displayed structural complexity. The heterogeneous structure imparts resistance and resilience to these forests and decreases susceptibility to future similar disturbance types [1].

The large horizontal and vertical heterogeneity of surviving regeneration is a foundation of high structural diversity within early-seral stands and is predicted to sustain such diversity through several decades of early-seral stand development [29]. The importance of post-disturbance spatial pattern of regeneration for future development increases with decreasing density of live residuals [5]. The structural heterogeneity evident in many primary Norway spruce forests thus serves an ecological function supporting biodiversity not only before, but also after disturbance.

The species composition of montane Norway spruce forests tends to remain unchanged [21], regardless of prevailing disturbance type—windthrow or bark beetle outbreak [17,59–60]. This differs from mixed forests in that wind or insect disturbances may be followed by spreading of tree species resistant to the disturbance agent [12,30,61].

Maintenance of rowan, which contributes forage and biodiversity functions in these high-elevation, spruce-dominated communities, is dependent on canopy-openings. Relatively few rowan trees were recruited post-disturbance, but survival of advance regeneration rowan was very high, and survivors exhibited higher, less variable height growth rates than spruce; this is partly because the distribution of rowan seedling height was more unimodal compared to the reverse-J distribution of spruce seedlings (Fig 3). Our results confirmed that renewal of rowan is primarily dependent on the release of an advance regeneration bank [52]. The recruitment of shade-tolerant rowan seedlings under mature canopies allows the formation of a seedling bank [51–52]. As the life span of rowan trees is shorter than the lifespan of spruce, rowan abundance decreases in the long-term absence of canopy-open disturbance [62], and may also act as a source of future canopy heterogeneity through death.

### Pre-disturbance structure influences post-disturbance early-seral structural development pathways

**Fig 6:**
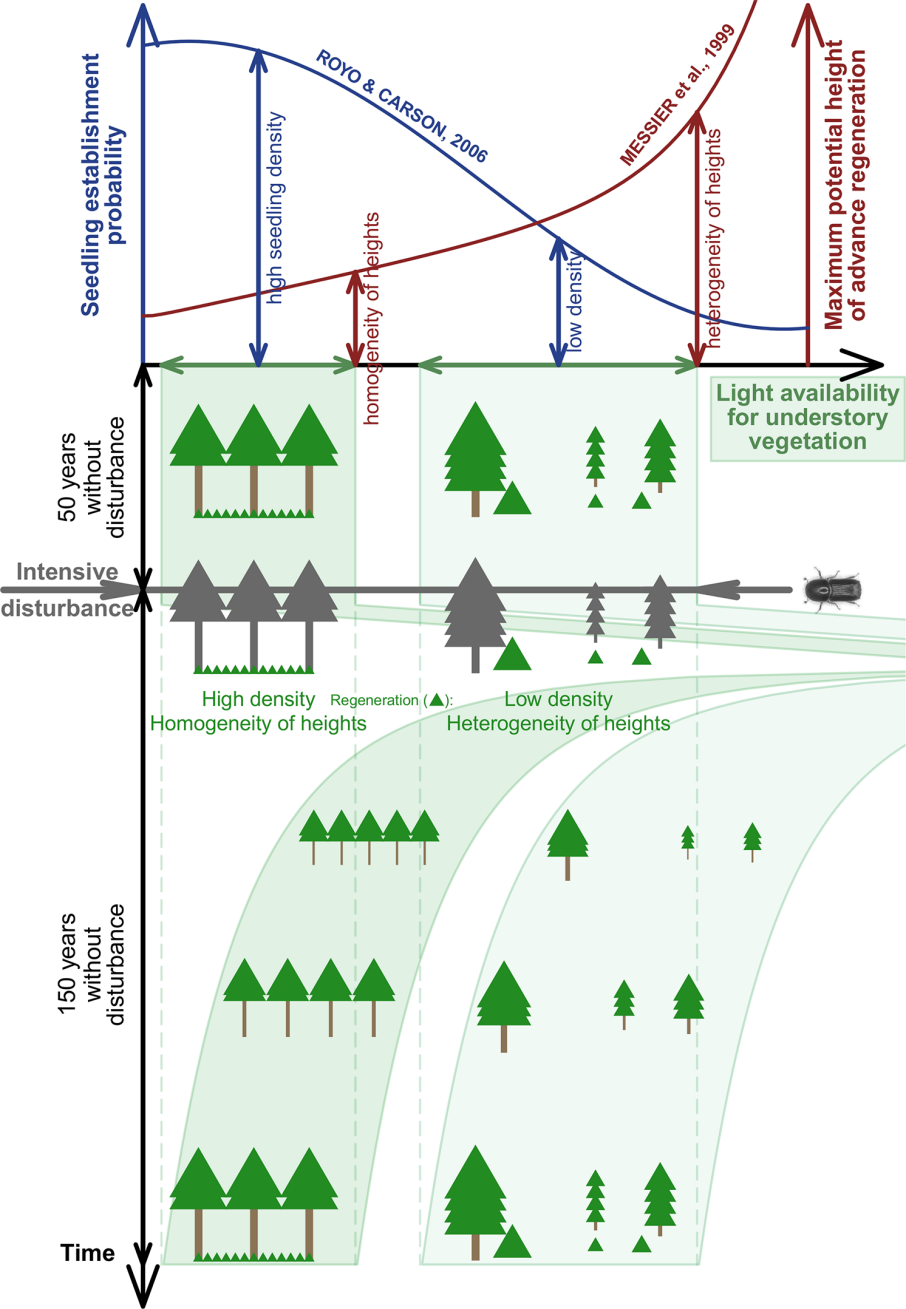
The conceptual figure illustrates how the structural complexity of an old-growth forest carries over to the next stand, in spite of a severe canopy-removing bark beetle disturbance. Structurally simple patches dominated by a dense overstory tend to be replaced by a young simple cohort (high density, more evenly spatially distributed, shorter and more uniform heights). Structurally complex patches that include gaps tend to be replaced by with a similarly complex young cohort (lower density, larger patches of open space, greater maximum and wider variability in heights). This difference in post-disturbance early-seral structural development pathways is attributed to light conditions and microsite availability when the advance regeneration bank was formed: Under densely closed canopies, there are suitable microsite conditions (sparse herb layer) for seedling establishment but not enough light for sustainable height growth over a certain threshold. In contrast, under moderate canopy cover, the establishment of new seedlings is inhibited by a dense herb layer. Seedlings and saplings that do manage to establish in these patches are subject to mortality due to resource competition by the herb layer, resulting in lower advance regeneration density, larger patches of open space and wider height variability under moderate canopy cover.

The Fig 6 illustrates how the structural complexity of an old-growth forest carries over to the next stand, in spite of a severe canopy-removing bark beetle disturbance. Structurally simple patches dominated by a dense overstory tend to be replaced by a young simple cohort (high density, more evenly spatially distributed, shorter and more uniform heights). Structurally complex patches that include gaps tend to be replaced by with a similarly complex young cohort (lower density, larger patches of open space, greater maximum and wider variability in heights). This difference in post-disturbance early-seral structural development pathways is attributed to light conditions and microsite availability when the advance regeneration bank was formed: Under densely closed canopies, there are suitable microsite conditions (sparse herb layer) for seedling establishment but not enough light for sustainable height growth over a certain threshold (~50 cm). Indeed, in a nearby stand with a more uniformly dense canopy, advance regeneration density was an order of magnitude greater than in our dense patches, with an even lower maximum height [20]. In contrast, under moderate canopy cover, the establishment of new seedlings is inhibited by a dense herb layer. Seedlings and saplings that do manage to establish in these patches are subject to mortality due to resource competition by the herb layer [23,58], resulting in lower advance regeneration density, larger patches of open space and wider height variability under moderate canopy cover.

Based on the above-described mechanism, the persistence of forest complexity within the post-disturbance stand could also be possible in mature stands with developed old-growth characteristics, such as a diversity of tree sizes and gaps in the forest canopy. As such, small- to meso-scale disturbances that break up dense canopies are important to both creating and perpetuating structural diversity across the forest sere.

## Implications and Conclusions

Heterogeneous stands encourage the development of heterogeneous regeneration patterns, thus, disturbances that minimize damage to the advance regeneration layer yields new stands with still-heterogeneous structural patterns; this mechanism acts as a memory of pre-disturbance structural patterns [8]. The logical converse of this observation is that structurally simple stands with a single dense canopy layer (e.g., managed second-growth in a stem-exclusion stage; [9]) would yield structurally simple stands when subject to bark beetle disturbance; this hypothesis could be verified in future research.

Increasingly, landscapes of Central Europe have become dominated by even-aged and even-density stands. Two centuries of management designed to increase the homogeneity of spruce forest structure have increased the susceptibility of forested landscapes to large-scale disturbance [1]. Forest stands with homogeneous canopies potentially diminish biodiversity by reducing understory light gaps and microenvironments [27]. Structurally complex forests may provide increased resistance and resilience to severe disturbances. Despite the perception that early-seral forests related to severe disturbance are structurally simple, high-severity disturbances common in high-elevation Norway spruce forests can produce early-seral forests with heterogeneous structure (sensu [26]). The wide spatial variability of regeneration associated with pre-disturbance structures represents a crucial biological legacy, which, in turn, is related to the previous disturbances that influenced stand structure. This memory of pre-disturbance structural patterns may explain why spruce-free patches may persist for several decades [23,55]. Prior canopy structure and persistence of microsite conditions (e.g. fern cover) can provide relative stability of forest structure complexity and spatial pattern, which may persist for several decades and even through multiple forest generations.

## Supporting Information

S1 Fig Sapling density as a function of A) canopy cover and B) Alpine-lady fern cover. Grey areas represent the 95% confidence envelopes of the density functions. The rasters of canopy or Alpine-lady fern cover (colour range from light green representing the lowest cover to violet representing the highest cover) are shown with the position of the saplings represented by black dots.
(TIF)

S2 Fig Relative distribution estimates of spruce sapling densities based on distance to the nearest down log. Logs were classified according to side vegetation cover. The grey-shaded areas indicate the envelope of uncertainty around the estimates of sapling density, as indicated by the differentiated lines.
(TIF)

S3 Fig Bivariate spatial pattern between survived and newly recruited spruces. The null model was constructed using random shifting. Positions of survived and newly recruited spruces (above 50 cm) are marked in subplot.
(TIFF)

S1 Table List of independent and supplementary variables used in PCA. (PDF)

S2 Table Tree and regeneration density before and after disturbance. The number (total for both plots) of survived undamaged, survived damaged, and dead spruce and rowan individuals by height classes (50–200 cm, > 200 cm) four years after the wind and bark beetle disturbance. Disturbance-related causes are in italics. The sign (-) means that *χ2* approximation of interspecific differences would be incorrect because the number of expected values is too low.
(PDF)
