# Supplementary figures and images for: Legacy of Pre-Disturbance Spatial Pattern Determines Early Structural Diversity following Severe Disturbance in Montane Spruce Forests
Source: PLoS One. 2015 Sep 30;10(9):e0139214. doi: 10.1371/journal.pone.0139214 (PMC4589365; doi:10.1371/journal.pone.0139214)

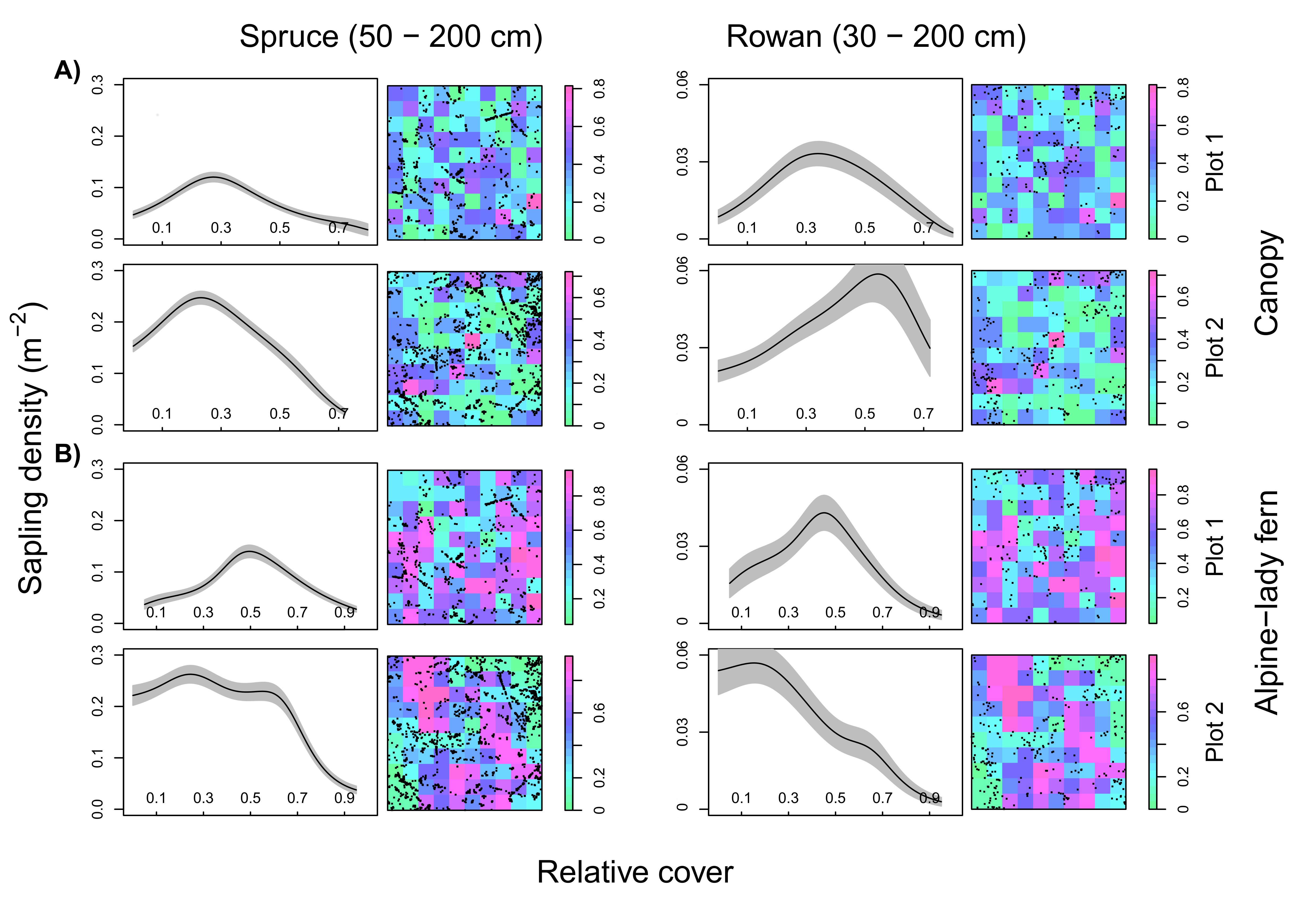

Supplement: S1 Fig — Grey areas represent the 95% confidence envelopes of the density functions. The rasters of canopy or Alpine-lady fern cover (colour range from light green representing the lowest cover to violet representing the highest cover) are shown with the position of the saplings represented by black dots. (TIF) [file pone.0139214.s001.tif]

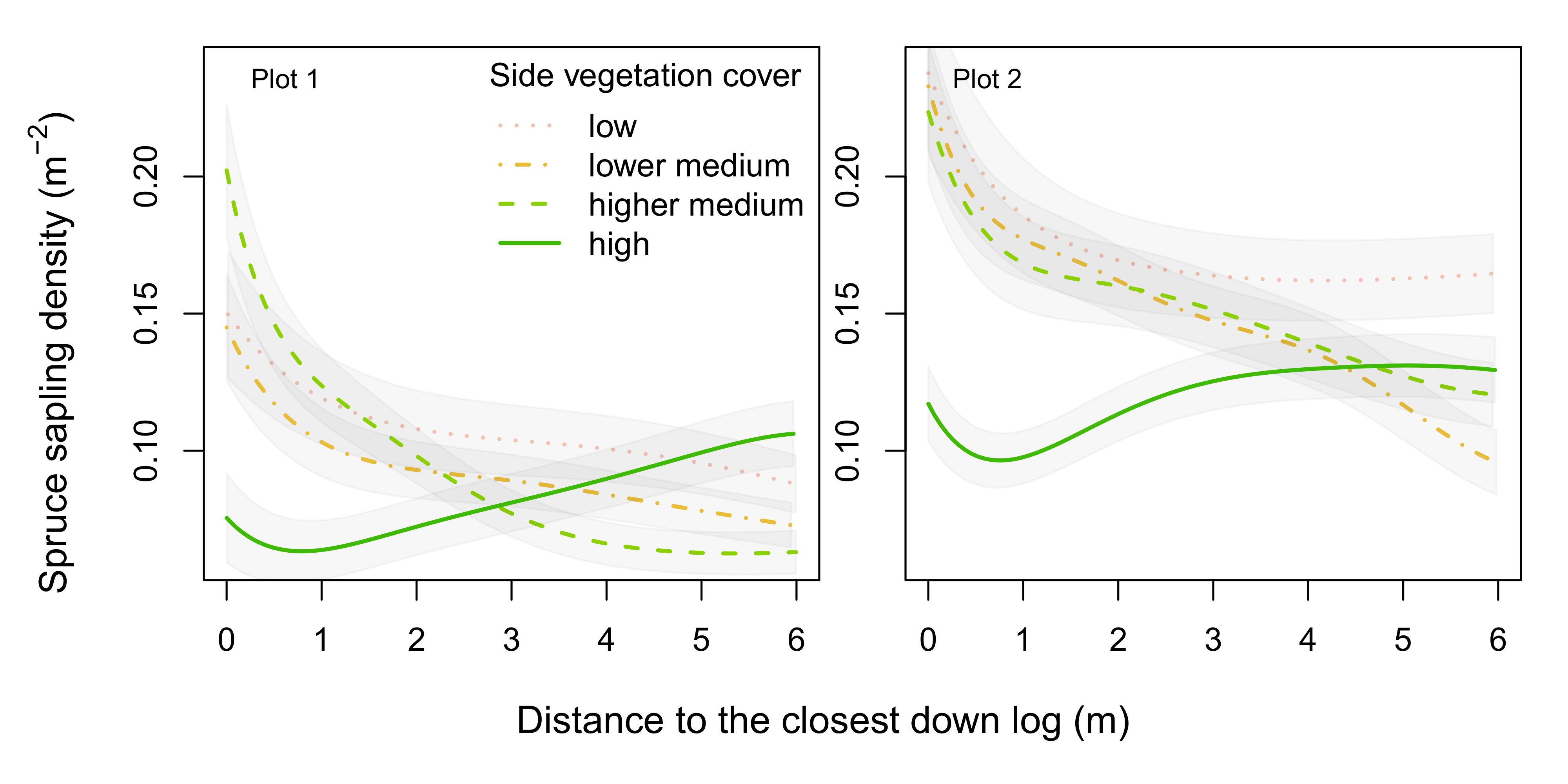

Supplement: S2 Fig — Logs were classified according to side vegetation cover. The grey-shaded areas indicate the envelope of uncertainty around the estimates of sapling density, as indicated by the differentiated lines. (TIF) [file pone.0139214.s002.tif]

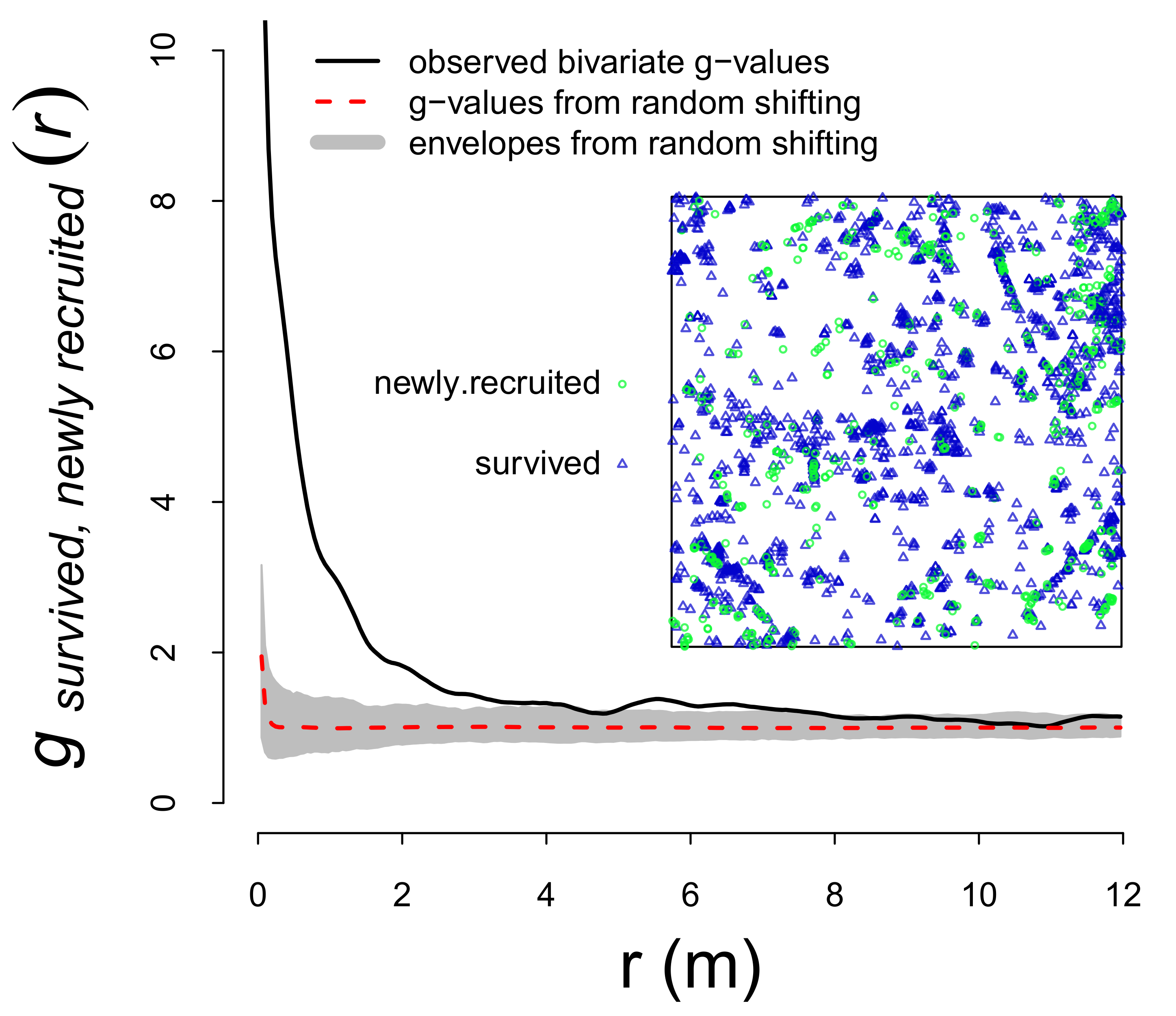

Supplement: S3 Fig — The null model was constructed using random shifting. Positions of survived and newly recruited spruces (above 50 cm) are marked in subplot. (TIFF) [file pone.0139214.s003.tiff]
