# Supplementary material for: Legacy of Pre-Disturbance Spatial Pattern Determines Early Structural Diversity following Severe Disturbance in Montane Spruce Forests
Source: PLoS One. 2015 Sep 30;10(9):e0139214. doi: 10.1371/journal.pone.0139214 (PMC4589365; doi:10.1371/journal.pone.0139214)
Supplement: S1 Table — (PDF) [file pone.0139214.s004.pdf]

Independent variables	Definition/Methods
Canopy cover per grid cell	Defined as 100% minus the area without crown projection
Alpine-lady fern cover per grid cell	The cover (dominance) was estimated. Values were recorded to the nearest 5%.
Distance to the closest down log	The perpendicular distance to the closest longitudinal axis of a down log
Side vegetation cover along down logs	The vegetation growing along a log and/or rising over the log): (1) up to 5% of the log covered; (2) 6 to 25% covered; (3) 26 to 50% covered and (4) >50% covered
**Supplementary variables in PCA**	
Density	Sapling density. Trees under 200 cm per ha
Height homogeneity	Pearson's measure of kurtosis of sapling height distribution
Height heterogeneity	Interquartile range of sapling height distribution
Canopy	Canopy cover per grid cell
Fern	Alpine-lady fern cover per grid cell

S1 Table. List of independent and supplementary variables used in PCA.
