# Supplementary material for: Legacy of Pre-Disturbance Spatial Pattern Determines Early Structural Diversity following Severe Disturbance in Montane Spruce Forests
Source: PLoS One. 2015 Sep 30;10(9):e0139214. doi: 10.1371/journal.pone.0139214 (PMC4589365; doi:10.1371/journal.pone.0139214)
Supplement: S2 Table — The number (total for both plots) of survived undamaged, survived damaged, and dead spruce and rowan individuals by height classes (50–200 cm, > 200 cm) four years after the wind and bark beetle disturbance. Disturbance-related causes are in italics. The sign (-) means that χ2 approximation of interspecific differences would be incorrect because the number of expected values is too low. (PDF) [file pone.0139214.s005.pdf]

			Norway Spruce		Rowan		Interspecific differences
	**Category**	**Reason**	**No.**	**% of total**	**No.**	**% of total**	**$\chi2$ value (p -value)**
	**Survived trees (> 200 cm)**		**161**		**3**		
	**Killed trees (> 200 cm)**	*bark beetle/windthrow*	**421**		**1**		
**Pre-disturbance saplings (50 – 200 cm)**	**Survived undamaged**		**1886**	**87.7%**	**147**	**29.7%**	**285.0 (≤ 0.001)**
	**Survived damaged total**		**168**	**7.8%**	**343**	**69.3%**	**346.4 (≤ 0.001)**
		bark beetle attack	13	0.6%	0	0.0%	(-)
		another death tree fall	8	0.4%	0	0.0%	(-)
		an. windthrow tree fall	59	2.7%	5	1.0%	81.3 (≤ 0.001)
		uprooting from deadwood	7	0.3%	0	0.0%	(-)
		other (snow, etc.)	77	3.6%	12	2.4%	88.1 (p≤ 0.001)
		browsing and bark stripping	4	0.2%	326	65.9%	124.2 (p≤ 0.001)
	**Killed total**		**96**	**4.5%**	**5**	**1.0%**	**21.6 (p≤ 0.001)**
		bark beetle attack	9	0.4%	0	0.0%	(-)
		another death tree fall	3	0.1%	0	0.0%	(-)
		an. windthrow tree fall	65	3.0%	5	1.0%	(-)
		uprooting from deadwood	3	0.1%	0	0.0%	(-)
		competition	10	0.5%	0	0.0%	(-)
		other (snow, etc.)	3	0.1%	0	0.0%	(-)
		browsing and bark stripping	3	0.1%	0	0.0%	(-)
	**Total pre-disturbance saplings**		**2414**	**100.0%**	**843**	**100.0%**	

S2 Table. Tree and regeneration density before and after disturbance. The number (total for both plots) of survived undamaged, survived damaged, and dead spruce and rowan individuals by height classes (50–200 cm, > 200 cm) four years after the wind and bark beetle disturbance. Disturbance-related causes are in italics. The sign (-) means that $\chi2$ approximation of interspecific differences would be incorrect because the number of expected values is too low.
